# Comparison of three non-invasive hemodynamic monitoring methods in critically ill children

**DOI:** 10.1371/journal.pone.0199203

**Published:** 2018-06-18

**Authors:** Chanapai Chaiyakulsil, Marut Chantra, Poomiporn Katanyuwong, Anant Khositseth, Nattachai Anantasit

**Affiliations:** 1 Department of Pediatrics, Division of Pediatric Critical Care, Department of Pediatric, Faculty of Medicine, Ramathibodi Hospital, Mahidol University, Bangkok, Thailand; 2 Department of Pediatrics, Division of Cardiology, Department of Pediatric, Faculty of Medicine, Ramathibodi Hospital, Mahidol University, Bangkok, Thailand; University of Bern, University Hospital Bern, SWITZERLAND

## Abstract

**Introduction:**

Hemodynamic parameters measurements were widely conducted using pulmonary artery catheter (PAC) with thermodilution as a reference standard. Due to its technical difficulties in children, transthoracic echocardiography (TTE) has been widely employed instead. Nonetheless, TTE requires expertise and is time-consuming. Noninvasive cardiac output monitoring such as ultrasonic cardiac output monitor (USCOM) and electrical velocimetry (EV) can be performed rapidly with less expertise requirement. Presently, there are inconsistent evidences, variable precision, and reproducibility of EV, USCOM and TTE measurements. Our objective was to compare USCOM, EV and TTE in hemodynamic measurements in critically ill children.

**Materials and methods:**

This was a single center, prospective observational study in critically ill children. Children with congenital heart diseases and unstable hemodynamics were excluded. Simultaneous measurements of hemodynamic parameters were conducted using USCOM, EV, and TTE. Inter-rater reliability was determined. Bland-Altman plots were used to analyse agreement of assessed parameters.

**Results:**

Analysis was performed in 121 patients with mean age of 4.9 years old and 56.2% of male population. Interrater reliability showed acceptable agreement in all measured parameters (stroke volume (SV), cardiac output (CO), velocity time integral (VTI), inotropy (INO), flow time corrected (FTC), aortic valve diameter (AV), systemic vascular resistance (SVR), and stroke volume variation (SVV); (Cronbach’s alpha 0.76–0.98). Percentages of error in all parameters were acceptable by Bland-Altman analysis (9.2–28.8%) except SVR (30.8%) and SVV (257.1%).

**Conclusion:**

Three noninvasive methods might be used interchangeably in pediatric critical care settings with stable hemodynamics. Interpretation of SVV and SVR measurements must be done with prudence.

## Introduction

Measurement of cardiac output is crucial and provides important information in assessment of hemodynamics in critically ill patients [1–5]. In the past, assessment was done invasively using pulmonary artery catheter (PAC) with thermodilution technique and has served as reference standard over the years [3–9]. Nevertheless, the technique is difficult and rarely feasible in children due to limited catheter size and small vessel. Furthermore, it posed possible complications such as infection and thromboembolic events even in adult studies [6, 10–14]. Therefore, Doppler studies such as transthoracic echocardiography (TTE) or transesophageal echocardiography (TEE) has been widely used for hemodynamic measurements and has been validated to be equivocal with PAC thermodilution with acceptable validity [3, 13–18]. Consequently, TTE has replaced PAC with thermodilution in clinical settings. Nonetheless, the process is time consuming, requiring expertise and cannot be continuously monitored. With the requirement of expertise and time, it is difficult to be conducted in remote areas without cardiologist and also in critical setting where accurate judgments and managements must be done. There are recent developments of noninvasive cardiac output monitoring, such as ultrasonic cardiac output monitor (USCOM) and impedance cardiography (electrical velocimetry; EV) which can be done rapidly in the critical setting and requiring less expertise. USCOM uses Doppler ultrasound to measure the velocity of blood passing through the aortic or pulmonary valve in order to calculate the cardiac output [3–5, 19–23]. EV transcutaneously detects changes in impedance with changes in erythrocyte orientation and flow peak velocity in the ascending aorta for the continuous quantification of cardiac output [19–21, 24–25]. Both recent developments can be performed simply by non-cardiologists, which serve as simpler tools for rapid evaluation of patients in areas without cardiologist. With current studies in both pediatrics and adults, it was difficult to demonstrate the validity of other non-invasive techniques in assessment of hemodynamic parameters, such that there were inconsistent evidence and variable precision and reproducibility of EV and USCOM when compared with standard echocardiography or PAC thermodilution [2–6, 22–27]. To our knowledge, no study has been done to compare all the three methods (USCOM, EV and TTE) in measurement of hemodynamic parameters in critically ill children. Our objective was to compare the variability and validity of the three non-invasive hemodynamic monitoring machines. We expected comparable results of measurements between all the three modalities. Thus, each method could be used interchangeably in critical settings without availability of cardiologists.

## Materials and methods

This study was approved by the committee on human right related to research involving human subjects, Faculty of Medicine Ramathibodi Hospital, Mahidol University and all participants’ parent or guardian provided informed written consent. It was a prospective, observational study comparing USCOM, EV, and TTE for measurement of hemodynamic parameters in critically ill children. The study was performed at a pediatric intensive care unit in a tertiary care academic center. It is a regional referral center for pediatric subspecialty care in Thailand.

Critically ill children, aged 1 month to 18 years who were admitted with critically ill conditions from September 2016 to June 2017 were screened for enrollment. Decision of admission were made by proxy of severity of illness and based on the decision of attending pediatric intensivist. The exclusion criteria were children with congenital heart diseases, unstable hemodynamics, showing excessive discomfort during study, burns involving area of impedance electrodes placement and severe pulmonary disease obscuring echocardiography window. Patient with unstable hemodynamics was defined as a patient with hypotension for age and those who required inotropic adjustment within 6 hours before measurements. The sample size was calculated by using Bland-Altman formula with standard error of 95% confidence interval of limit agreement, which is approximately root (3s^2^/n). Since no previous study was conducted for comparison of the three methods, 30% of percentage error of agreement was used as precision criterion for sample size calculation based on prior study in PAC with thermodilution [28]. Approximately 120 participants would suffice with acceptable precision.

### Study protocol

Three noninvasive hemodynamic monitoring machines were USCOM (Sydney, Australia), ICON (EV; Osypka Medical, Germany), and transthoracic echocardiography (Philips iE33, USA). USCOM and ICON measurements were obtained by a pediatric intensivist who was blinded from the simultaneous data by using three data recorders. Twenty patients were recruited to test the inter-rater reliability. The inter-rater reliability was determined using Cronbach’s alpha. Transthoracic echocardiography was performed by well-trained echocardiographers. After obtaining acceptable reliability, all measurements were taken simultaneously for at least 3 cardiac cycles and recorded by three individuals and results were blinded. USCOM data were measured first and then TTE data were measured within 5 minutes of time frame between each measurement. Measurements were obtained twice within 30 minutes to ensure precision and the average was used for analysis. Demographic data, admission diagnosis, vital signs, ventilator support, inotropic support, mortality, duration of PICU stay and hospital stay were collected.

Hemodynamic parameters obtained from all the three non-invasive machines were categorized into three groups: preload, afterload and overall results of cardiac output and cardiac index. We measured aortic valve (AV) diameter and velocity time integral (VTI) using Doppler with TTE to obtain stroke volume. In USCOM, by using patient’s age, weight and height, AV diameter was calculated. USCOM, similar to that of TTE, also used Doppler to measure VTI. With available AV diameter and VTI, stroke volume could be obtained using USCOM. In context of preload; stroke volume variation (SVV) is defined as stroke volume which is variation by respiratory cycle, corrected flow time (FTC) is the systole time divided by the square root of cardiac cycle time, which quantifies the time of blood flow through aortic valve. By incorporating heart rate with SV, we can measure cardiac output (CO) and cardiac index (CI). With the input of blood pressure, afterload in the form of systemic vascular resistance (SVR) and systemic vascular resistance index (SVRI) can be computed. As mentioned earlier, ICON measured the impedance of erythrocytes and flow velocity across the aortic valve to quantify cardiac output and cardiac index as well as the afterload. Measured parameters were demonstrated in Table 1.

**Table 1:** Hemodynamic parameters were categorized by preload, afterload and others.

Parameters	USCOM	ICON	2D-Echocardiogram
Preload	Stroke volume (SV) Stroke volume variation (SVV) Corrected flow time (FTC)	Stroke volume (SV) Stroke volume variation (SVV) Corrected flow time (FTC)	Stroke volume
Afterload	Systemic vascular resistance (SVR) Systemic vascular resistance index (SVRI)	Systemic vascular resistance (SVR) Systemic vascular resistance index (SVRI)	-
Other parameters	Aortic valve diameter (AV) Velocity time integral (VTI) Cardiac output (CO) Cardiac index (CI)	Cardiac output (CO) Cardiac index (CI)	Aortic valve diameter (AV) Velocity time integral (VTI) Cardiac output (CO) Cardiac index (CI)

### Statistical analyses

Continuous data were described as mean and standard deviation. Bland-Altman plots and percentage error were used to analyze agreement of assessed parameters. Statistics were performed with SPSS version 17.0 (IBM corporation, Armonk, New York) and MedCalc version 16.4.3 (Ostend, Belgium). Percentages of error >30% were considered statistically significant error [29].

## Results

**Fig 1:**
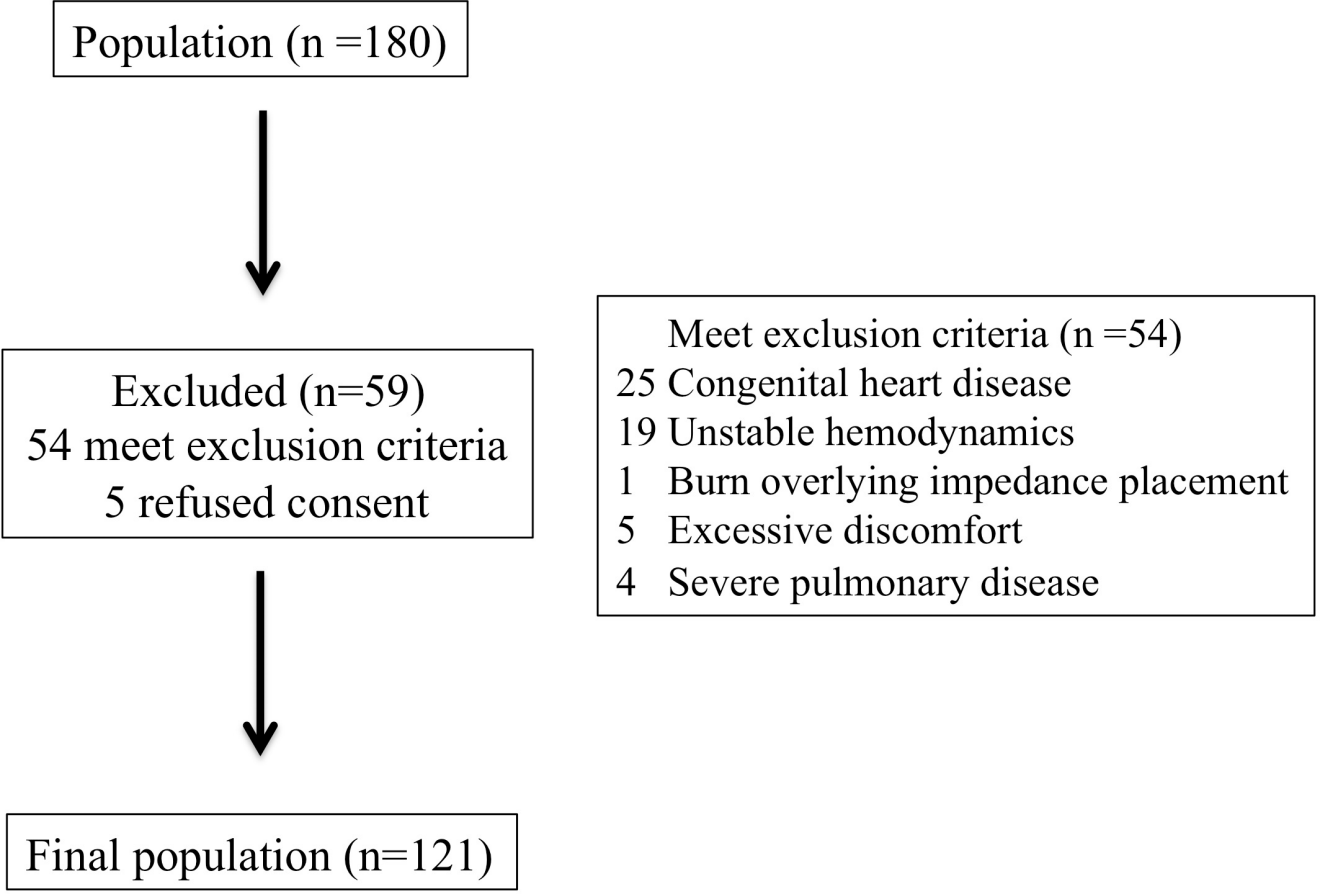
Flow diagram showing the screening and enrollment of patients.

The average inter-rater reliability of measured parameters using Cronbach’s alpha were 0.76 for USCOM, 0.92 for ICON and 0.98 for echocardiogram. We screened 180 patients who were admitted during the study period. Total of 54 patients met the exclusion criteria and 5 refused consent (Fig 1). Total of 121 participants were enrolled with total of 726 measurements, 56% were male and mean age was 4.9 years old. We found 82 (68%) patients with comorbidities with majority being neurologic (mainly epilepsy) (20.7%) and gastrointestinal (mainly biliatry atresia) (15.7%). The mortality rate for overall hospital stay was 4.1%. Non-survivors had significantly higher BSA, rate of inotropic and sedation utilization with longer hospital stay (Table 2).

**Table 2:** Baseline characteristics of patients who were admitted with critically ill conditions.

Clinical characteristics	Participants (n = 121)	Non-survivors (n = 5)	Survivors (n = 116)
Gender: male, n (%)	68 (56.2)	2 (40)	66 (56.9)
Age (years)	4.9 ± 4.6	8 ± 7.3	4.9 ± 4.8
Weight (kg)	19.8 ± 16.2	33.6 ± 26	19.3 ± 15.6
Body surface area (m^2^)*	0.73 ± 0.39	1.02 ± 0.65	0.71 ± 0.37
Indication of admission, n (%)			
- Respiratory	37 (30.6)	1 (20.0)	36 (31)
- Postoperative	29 (24.0)	1 (20.0)	28 (24.1)
- Neurologic	23 (19.0)	1 (20.0)	22 (19.0)
- Infection (sepsis)	11 (9.1)	1 (20.0)	10 (8.6)
- Others	21 (17.3)	1 (20.0)	20 (17.3)
Comorbidities, n (%)			
- Healthy	39 (32.2)	1 (20.0)	38 (32.8)
- Neurologic	25 (20.7)	1 (20.0)	24 (20.7)
- Gastrointestinal	19 (15.7)	2 (40.0)	17 (14.6)
- Oncology	14 (11.6)	1 (20.0)	13 (11.2)
- Respiratory	11 (9.1)	0 (0.0)	11 (9.5)
- Others	13 (10.7)	0 (0.0)	13 (11.2)
Sedation used, n (%)*	20 (16.5)	4 (80.0)	17 (14.7)
Neuromuscular blockage used, n (%)	3 (2.5)	0 (0.0)	3 (2.6)
Inotropic drugs used, n (%)*	13 (10.7)	3 (60.0)	10 (8.6)
Respiratory support, n (%)			
- None	36 (29.8)	1 (20.0)	35 (30.2)
- Invasive MV	38 (31.4)	2 (40.0)	36 (31.0)
- NIPPV	5 (4.1)	0 (0.0)	5 (4.3)
- Low flow oxygen cannula	32 (26.4)	2 (40.0)	30 (25.9)
- High flow nasal cannula	10 (8.3)	0 (0.0)	10 (8.6)
Outcomes			
- Length of ICU stay (days in range)	8.2 (1–42)	5.2 (2–10)	8.4 (1–46)
- Length of hospital stay (days in range)*	26.6 (2–222)	67.7 (2–222)	24.5 (2–174)
- Mortality, n (%)	5 (4.1)		

*P-value < 0.05 for comparison of survivors and non-survivors

NIPPV: noninvasive positive pressure ventilation

### Bland Altman analysis results

We compared AV diameter and VTI obtained from USCOM and TTE. Mean AV diameter obtained from USCOM and TTE were 1.28 ± 0.66 cm and 1.36 ± 0.66 cm, respectively. Mean VTI measured by USCOM was 18.6 ± 8.2 cm, while measuring with TTE resulted as 18.7 ± 9.6 cm. By using TTE as gold standard, mean AV diameter difference and VTI difference measured by USCOM were -0.1 ± 0.2 cm and -0.1 ± 4.2 cm, respectively. Percentages of error were 9.2% and 12.7% for AV diameter and VTI, respectively.

**Fig 2:**
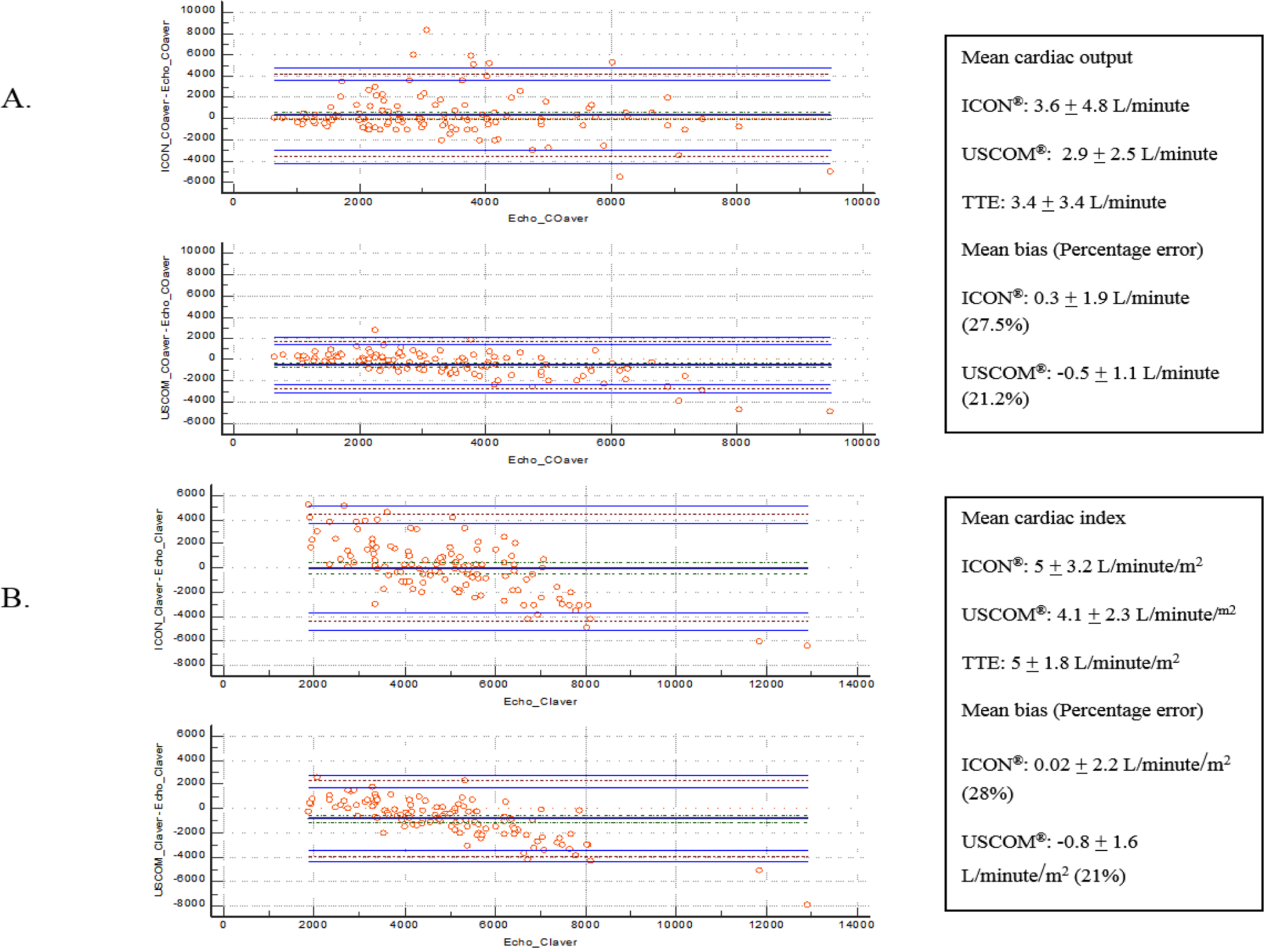
Bland-Altman analysis of overall results: Cardiac output (A), cardiac index (B). X-axis illustrated average measurements of standard from using TTE. Y-axis demonstrated mean bias using other modalities.

Mean cardiac output illustrated by USCOM, ICON and TTE were 2.9 ± 2.5 L/minute, 3.6 ± 4.8 L/minute and 3.4 ± 3.4 L/minute, respectively. Again using TTE as standard for three methods comparison, ICON illustrated cardiac output mean bias of 0.3 ± 1.9 L/minute while USCOM illustrated mean bias of -0.5 ± 1.1 L/minute with percentage of error of 27.5% for ICON and 21.2% of USCOM. In terms of cardiac index, ICON demonstrated mean cardiac index of 5 ± 3.2 L/minute/m^2^, 4.1 ± 2.3 L/minute/m^2^ for USCOM and 5 ± 1.8 L/minute/m^2^ for TTE. ICON illustrated cardiac index mean bias of 0.02 ± 2.2 L/minute/m^2^ while USCOM demonstrated mean bias of -0.8 ± 1.6 L/minute/m^2^ with percentages of error of 28% for ICON and 21% of USCOM (Fig 2).

### Preload

**Fig 3:**
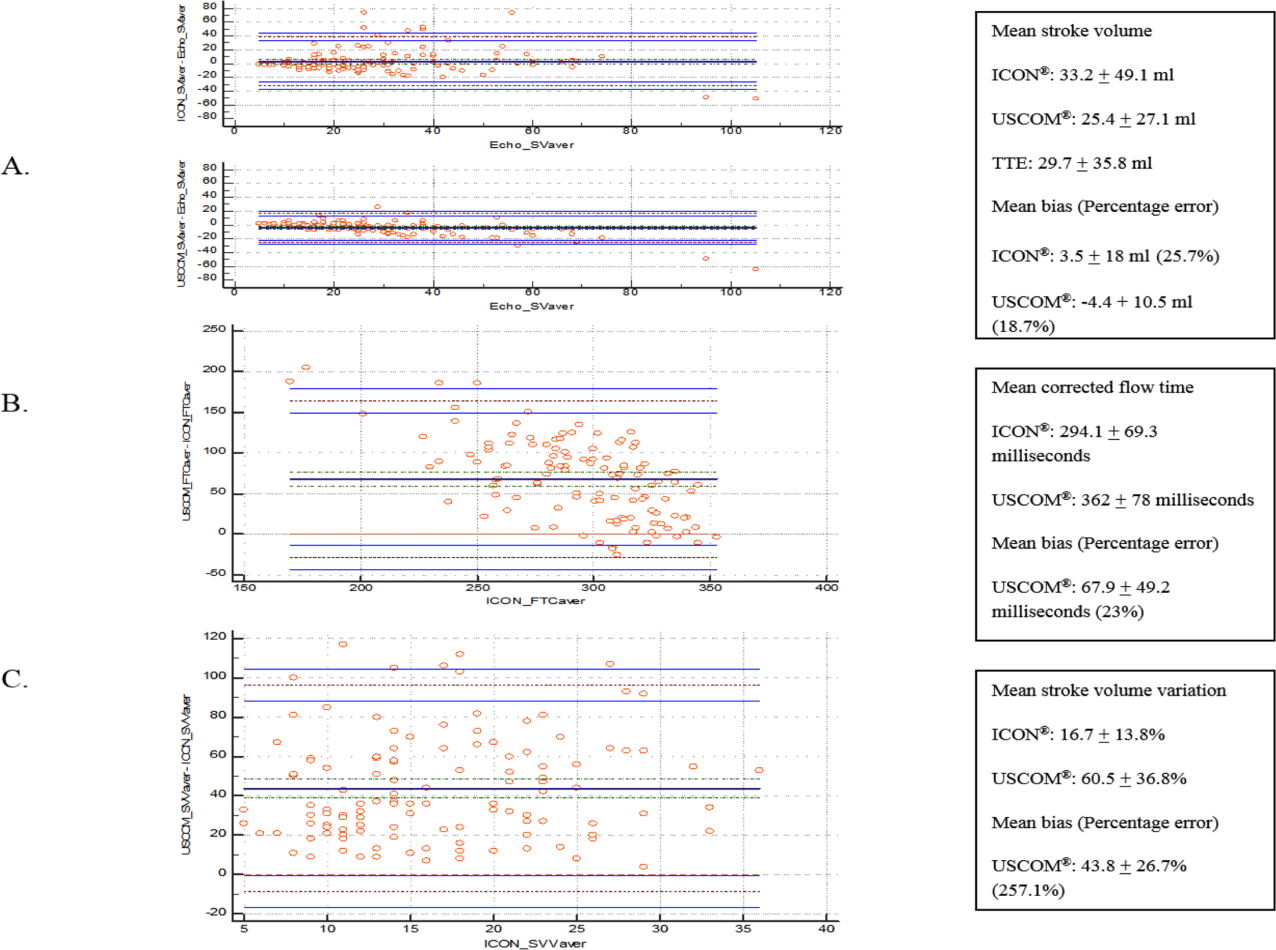
Bland-Altman analysis of preload: Stroke volume (A), Corrected flow time (B) and Stroke volume variation (C). X-axis illustrated average measurements of standard from using TTE or ICON. Y-axis demonstrated mean bias using other modalities.

Mean SV demonstrated by ICON was 33.2 ± 49.1 ml, USCOM was 25.4 ± 27.1 ml and TTE was 29.7 ± 35.8 ml. Using TTE as standard, SV mean bias was 3.5 ± 18 ml for ICON and -4.4 ± 10.5 ml for USCOM. Percentage errors were 25.7% and 18.7% for ICON and USCOM, respectively. Measurement of mean FTC and SVV for ICON was 294.1 ± 69.3 milliseconds and 16.7 ± 13.8%, respectively. On the other hand, using USCOM, mean FTC and SVV were 362 ± 78 milliseconds and 60.5 ± 36.8%, respectively. In analysis of FTC and SVV, ICON served as standard due to better inter-rater reliability and less operator dependent. Mean bias for FTC was 67.9 ± 49.2 milliseconds and for SVV was 43.8 ± 26.7%. USCOM showed percentages error of 23% for FTC and 257.1% for SVV (Fig 3).

### Afterload

**Fig 4:**
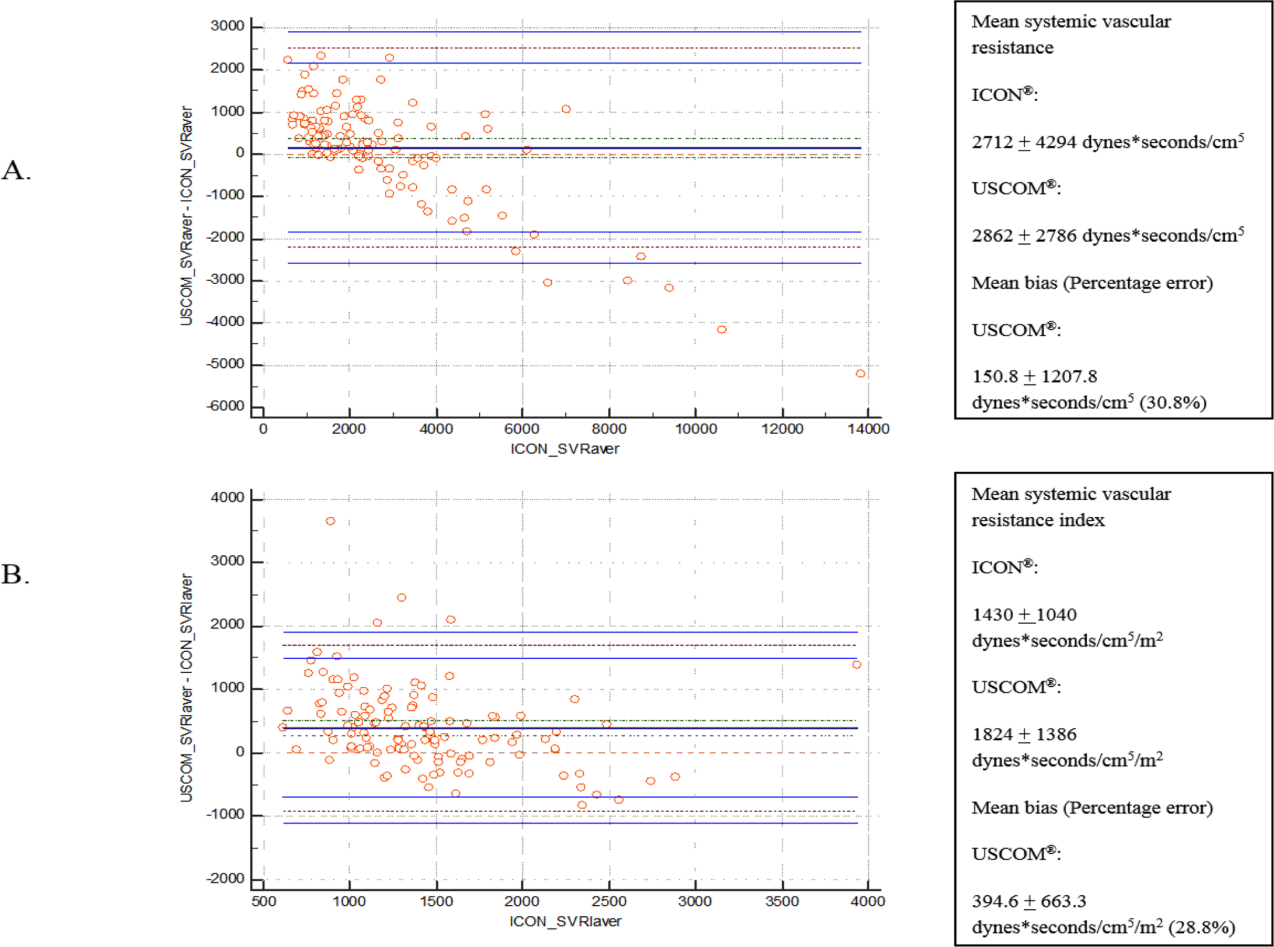
Bland-Altman analysis of afterload: Systemic vascular resistance (A) Systemic vascular resistance index (B). X-axis illustrated average measurements of standard from using ICON. Y-axis demonstrated mean bias using USCOM.

Mean SVR and SVRI for ICON were 2712 ± 4294 dynes*seconds/cm^5^ and 1430 ± 1040 dynes*seconds/cm^5^/m^2^, respectively. Using USCOM, mean SVR measured was 2862 ± 2786 dynes*seconds/cm^5^ while mean SVRI was 1824 ± 1386 dynes*seconds/cm^5^/m^2^. ICON was considered as standard for analysis of SVR and SVRI. Analysis revealed mean bias of 150.8 ± 1207.8 dynes*seconds/cm^5^ for SVR and mean bias of 394.6 ± 663.3 dynes*seconds/cm^5^/m^2^ for SVRI. Percentages of error were 30.8% for SVR and 28.8% for SVRI (Fig 4). Bland-Altman results for each parameter including mean measurements, mean bias, and percentage were again depicted in Table 3.

**Table 3:** Bland-Altman results summary.

Parameters	USCOM (Mean)	ICON (Mean)	TTE (Mean)	Mean bias	Percentage Error (%)
Preload					
Stroke volume (SV) (ml) (TTE–Standard)	25.4 ± 27.1	33.2 ± 49.1	29.7 ± 35.8	ICON: 3.5 ± 18 USCOM: -4.4 ± 10.5	ICON: 25.7 USCOM: 18.7
Stroke volume variation (SVV) (%) (ICON–standard)	60.5 + 36.8%	16.7 + 13.8		43.8 + 26.7	257.1*
Corrected flow time (FTC) (milliseconds) (ICON–standard)	362 + 78	294.1 + 69.3		67.9 + 49.2	23
Afterload					
Systemic vascular resistance (SVR) (dynes*seconds/cm5/m2) (ICON–standard)	2862 + 2786	2712 + 4294		150.8 + 1207.8	30.8*
Systemic vascular resistance index (SVRI) (dynes*seconds/cm5/m2) (ICON–standard)	1824 + 1386	1430 + 1040		394.6 + 663.3	28.8
Other parameters					
Aortic valve diameter (AV) (cm) (TTE–standard)	1.28 + 0.66		1.36 + 0.66	-0.1 + 0.2	9.2
Velocity time integral (VTI) (cm) (TTE–standard)	18.6 + 8.2		18.7 + 9.6	-0.1 + 4.2	12.7
Cardiac output (CO) (L/minute) (TTE standard)	2.9 + 2.5	3.6 + 4.8	3.4 + 3.4	ICON: 0.3 + 1.9 USCOM: -0.5 + 1.1	ICON: 27.5 USCOM: 21.2
Cardiac index (CI) (L/minute) (TTE standard)	4.1 + 2.3	5 + 3.2	5 + 1.8	ICON: 0.02 + 2.2 USCOM: -0.8 + 1.6	ICON: 28 USCOM: 21

*Percentage error >30%

## Discussion

Hemodynamic monitoring is crucial in making appropriate decisions for managements of critically ill children. By having user-friendly, accurate and less time-consuming non-invasive hemodynamic monitoring methods, suitable interventions would result in less complications, morbidity and mortality. We aimed to compare and expect comparable measurement results between all the three non-invasive machines, USCOM, ICON and TTE. Therefore, each method can be used interchangeably in pediatric critical care settings.

To our knowledge, this study is one of the largest studies comparing non-invasive hemodynamic monitoring machines with total of 726 measurements and the only study to date which compared three non-invasive machines. Good inter-rater reliability was demonstrated throughout the parameters in all three machines (average Cronbach’s alpha 0.76–0.98). Using Bland-Altman analysis, study revealed acceptable percentage of error (<30%) throughout all parameters except SVR and SVV (30.8% and 257.1%, respectively). Our results were different from prior study in 2014 from our institution, which showed incomparable measurements between USCOM and TTE in septic shock pediatric patients [3]. It is probably due to different group of population in our study which included only hemodynamically stable patients. We demonstrated similar results with studies performed by previous studies which revealed comparable measurements between TTE and EV [24–26].

Most previous studies illustrated their results using only stroke volume and cardiac output as parameters and did not analyze the other measurable parameters within each method [3–5, 22–26]. In critical care setting, by incorporating all the parameters, the interpretation would be more accurate and aids in decision-making. Our study attempted to break down the parameters into preload, afterload and cardiac output that can give clinicians more confidence and prudence in interpretation of each parameter.

This study has some limitations. Firstly, as a single center, observational study, it might be difficult to generalize our results in different population settings. Furthermore, since only hemodynamically stable patients were included in the study, unstable patients may yield different results. Lastly, in young children, it might be difficult to obtain the accurate data in a single measurement using USCOM and TTE due to uncomfortable nature of the test; we overcame this by performing the test twice with inter-rater reliability to ensure reproducibility and precision.

### Conclusions

It is feasible to use these three noninvasive machines interchangeably in pediatric critical care settings in patients with stable hemodynamics. Prudence must be employed in interpretation of SVV and SVR measurements.

### Area of future research

Since our study only demonstrated comparable results in hemodynamically stable patients, it is of great interest to conduct another study, especially in multi-center setting, or in patients with unstable hemodynamics.
